# Unmasking fluid overload in children on peritoneal dialysis: a multimodal diagnostic approach

**DOI:** 10.1007/s00467-025-06825-y

**Published:** 2025-07-03

**Authors:** Bahriye Atmis, Ikbal Turker, Derya Cevizli, Cagla Cagli Piskin, Faruk Ekinci, Dincer Yildizdas, Aysun K. Bayazit

**Affiliations:** 1 Department of Pediatric Nephrology, Faculty of Medicine, Çukurova University, Adana, Türkiye; 2 Department of Pediatric Intensive Care, Faculty of Medicine, Çukurova University, Adana, Türkiye

**Keywords:** Bioimpedance spectroscopy, Fluid status, Lung ultrasound, Overhydration, Peritoneal dialysis

## Abstract

**Background:**

This study aimed to assess fluid status in pediatric patients on peritoneal dialysis by combining ultrasonography and bioimpedance spectroscopy (BIS). It specifically focused on examining the changes in volume status following a 2-h dwell time ultrafiltration exchange and evaluating the reliability of these techniques.

**Methods:**

Thirteen pediatric patients on peritoneal dialysis were enrolled in this study, and their hydration status was assessed clinically. In addition, 56 lung ultrasound measurements, inferior vena cava (IVC) collapsibility index assessments, and BIS evaluations were performed both before and after a 2-h dwell exchange using 2.27%/2.5% dextrose dialysate.

**Results:**

The mean age of the patients was 8.6 ± 4.1 years, and eight of them (61.5%) were male. The IVC collapsibility index significantly increased (26.3 ± 10.0% vs. 44.4 ± 9.4%; *p* < 0.001), and the total number of B-lines significantly decreased (median 22 vs. 11.5; *p* < 0.001) after a 2-h dwell exchange using 2.27%/2.5% dextrose dialysate. A positive correlation was observed between the total number of B-lines and fluid overload measured using BIS both pre-dialysis (*r* = 0.504, *p* = 0.006) and post-dialysis (*r* = 0.528, *p* = 0.004). A significant reduction in the total number of B-lines was observed across all hydration groups after dialysis (*p* < 0.001). The area under the receiver-operating characteristic curve (AUC) for the total number of B-lines in predicting severe overhydration was 0.685 (*p* = 0.097) when assessed using BIS and 0.740 (*p* = 0.181) when assessed by weight.

**Conclusion:**

Our results highlight marked changes in fluid status parameters from pre- to post-dialysis, underscoring the clinical value of combining lung ultrasonography and BIS for monitoring fluid overload in pediatric patients undergoing peritoneal dialysis.

**Graphical abstract:**

**Figure:**
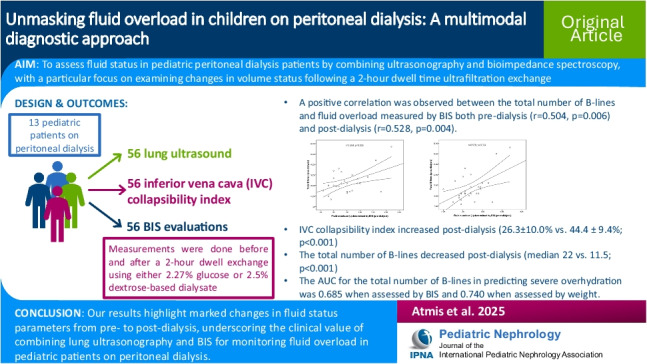
A higher resolution version of the Graphical abstract is available as Supplementary information

**Supplementary Information:**

The online version contains supplementary material available at 10.1007/s00467-025-06825-y.

## Introduction

Assessment of fluid status is challenging in children undergoing peritoneal dialysis due to the inadequacy of the clinical examination to reflect fluid status. Fluid overload is one of the common complications in children receiving kidney replacement therapy; it increases morbidity and mortality in children on peritoneal dialysis [1, 2]. Fluid overload in patients on dialysis usually leads clinically to hypertension and edema. Besides traditional methods such as clinical examination (blood pressure measurement, presence of lung crackles, edema, or jugular vein distension) and weight monitoring, accurate assessment of fluid overload using advanced methods has gained importance in recent years. Various methods, including bioimpedance spectroscopy (BIS), inferior vena cava (IVC) diameter and the derived collapsibility index, relative blood volume monitoring, N-terminal pro-brain natriuretic peptide (NT-proBNP), and lung ultrasound, have been used to investigate fluid overload in patients on dialysis [3–7].

Several studies have reported on assessing fluid overload in children on peritoneal dialysis, but an optimal assessment method is still lacking [8, 9]. In addition, children are in the growth phase, and hence, continuous weight gain causes difficulties in assessing fluid overload. Therefore, standardized, reliable methods are needed to accurately assess fluid overload, aiming to improve clinical outcomes and quality of life in pediatric patients on dialysis.

This study aimed to evaluate the fluid status in children on peritoneal dialysis by ultrasonography and BIS, focusing on demonstrating the correlation between these two methods.

## Materials and methods

### Patients

This prospective observational study included 13 pediatric patients on peritoneal dialysis at Balcalı Hospital, Çukurova University. The characteristics of the patients, including age, weight, height, vital signs (heart rate and blood pressure), dialysis modality, primary disease causing chronic kidney disease (CKD), and use of anti-hypertensive drugs, were recorded. Patients were evaluated during outpatient clinic visits, immediately before and after a 2-h dwell exchange using either 2.27% glucose or 2.5% dextrose-based dialysate, depending on the brand of peritoneal dialysis solution routinely used in their individual treatment regimen. With this procedure, we aimed to remove fluid by dialysis using a 2-h dwell time with moderately concentrated glucose peritoneal dialysis fluid. In patients on continuous ambulatory peritoneal dialysis (CAPD), the 2-h dwell dialysis was performed at the time of their routine exchange. In patients on automated peritoneal dialysis (APD), it was performed within a few hours after the completion of their night-time treatment during clinic visits. The hydration status of the patients was clinically assessed through weight and blood pressure measurements. Furthermore, 28 pre-dialysis and 28 post-dialysis lung ultrasounds, IVC collapsibility index assessments, and BIS measurements were performed.

Informed consent was obtained from the participants and/or guardians as specified in the ICMJE recommendations. The exclusion criteria were as follows: age older than 18 years, unwillingness to participate in the study, a duration of peritoneal dialysis of less than 3 months, and presence of an active infection. This study was approved by the Çukurova University Faculty of Medicine Non-Interventional Clinical Research Ethics Committee (Approval no.: 05.11.2021–116/4).

### Bioimpedance spectroscopy measurements

Multifrequency BIS was performed using a body composition monitor (Fresenius Medical Care, Hamburg, Germany). The device was calibrated and tested by clinical engineers before use. This equipment estimated total body water, intracellular and extracellular water, and overhydration by measuring impedance at 50 different frequencies ranging from 5 to 1000 kHz. The signals were delivered via the distal electrode and recorded by the proximal electrode [10]. A pediatric nephrology fellow (DC) received specialized training to perform BIS measurements. The patients were positioned supine during the procedure and made to rest without any physical contact with others. Weight-appropriate electrodes were used, with pediatric electrodes applied for patients weighing less than 20 kg. These electrodes were placed on the dorsal surfaces of the wrists and ankles, specifically on the same side of the body. The BIS measurements were conducted at the bedside shortly after the ultrasonographic evaluations.

The relative hydration score was determined using the following formula: (pre-dialysis body weight—normohydration weight)/extracellular water). Patients were classified into three relative hydration (RH) groups based on their fluid overload percentage: normal hydration (− 7 to 7%), moderate overhydration (7 to 15%), and severe overhydration (≥ 15%) [10–12].

### Ultrasonographic measurements

Ultrasonographic examinations were conducted at the bedside using a portable device (Resona7; Mindray Bio-Medical Electronics Co., Ltd., China), as described in our previous study [10]. The measurements were performed by one of our investigators (IT), who was skilled in bedside ultrasonography and had completed more than 250 such assessments. Lung ultrasound was performed using an L14-6 s linear probe with a frequency range of 5.1–12.5 MHz. The total number of B-lines was recorded via bedside ultrasonography to evaluate fluid overload. The thorax was divided into six zones on each side, with the B-lines counted in each zone. The sum of the B-lines across the 12 zones represented the total number of B-lines. Assessments were made with the patient in the supine position through the midclavicular and mid-axillary lines, from the second to the fifth intercostal spaces. The evaluations were made in the sitting position on the mid-scapular line, from the second to the fifth intercostal spaces.

The IVC diameter was measured with a C60n convex probe (2–5 MHz). The IVC diameter in the subxiphoid region was measured 1–2 cm distal to the junction of the hepatic vein junction during the breathing cycle using M-mode. The maximum IVC refers to the IVC diameter during normal expiration, whereas the minimum IVC refers to the IVC diameter during normal inspiration. The IVC collapsibility index was calculated by determining the percentage decrease in the IVC diameter from expiration to inspiration, using the formula: [max IVC − min IVC]/max IVC × 100%. The ultrasonographer was blinded to dry and current body weights, blood pressure, amount of ultrafiltration, and BIS measurements of the patients. All ultrasonographic evaluations were performed immediately before and after a 2-h dwell exchange using 2.27% glucose or 2.5% dextrose-based dialysate.

### Statistical analysis

Continuous variables with normal distribution were summarized as the mean and standard deviation, and those with non-normal distribution as the median and 25 th and 75 th percentile or interquartile range (IQR). The normality of distribution for continuous variables was confirmed using the Shapiro–Wilk test. Categorical variables were expressed as numbers and percentages. The Wilcoxon test or the paired *t*-test was used where appropriate for comparing of continuous variables between two related groups. The correlations between numeric variables that are distributed normally and non-normally were evaluated using Pearson simple correlation coefficients and the Spearman rank correlation coefficient, respectively. Receiver-operating characteristic (ROC) curve analysis was conducted to evaluate the accuracy of various variables in classifying patients with overhydration, and the area under the ROC curve (AUC) was determined. The statistical significance level was considered as 0.05 in all tests. The SPSS Statistics v.20.0 software package (IBM) was used to analyze the data statistically.

## Results

### Patient characteristics

The study included 13 children on peritoneal dialysis. The mean age of the patients was 8.6 ± 4.1 years, and eight of them (61.5%) were male. Nine patients (69.2%) were on APD, and four (30.8%) were on CAPD. The mean duration of peritoneal dialysis of the patients was 20.5 ± 18.3 months. The primary cause of CKD was glomerular diseases in five patients (38.5%), congenital anomalies of kidney and urinary tract (CAKUT) in four patients (30.8%), metabolic diseases in two patients (15.4%), and unknown cause of CKD was present in two patients (15.4%). Eleven patients (84.6%) received at least one anti-hypertensive drug. In 28 pre-dialysis measurements, 9 (32.1%) revealed normal hydration, 6 (21.4%) moderate overhydration, and 13 (46.5%) severe overhydration.

### Fluid status assessment and clinical findings

We performed BIS and ultrasound measurements in 13 patients, with 28 pre-dialysis and 28 post-dialysis measurements. The post-dialysis weight, systolic, and diastolic blood pressure measurements of the patients were found significantly lower than the pre-dialysis measurements (*p* < 0.001, *p* = 0.012, and *p* = 0.020, respectively). The median fluid overload based on dry weight was 3.15% (IQR: 4.67) pre-dialysis and 1.25% (IQR: 3.35) post-dialysis (*p* < 0.001). A statistically significant increase in the IVC collapsibility index (26.3 ± 10.0% vs. 44.4 ± 9.4%) (*p* < 0.001) was observed after a single dialysis exchange. In addition, a statistically significant decrease in the total number of B-lines (median 22 vs. 11.5) (*p* < 0.001) was noted after a single dialysis exchange. The internal jugular vein diameter, measured during both systole and diastole, did not significantly differ between pre- and post-dialysis measurements. A comparison of the pre- and post-dialysis measurements is presented in Table 1.

**Table 1:** Comparison of the pre- and post-dialysis measurements

Measurements	Pre-dialysis	Post-dialysis	*p*
Weight (kg), median (25p–75p)	25.2 (16.3–33.3)	25.1 (15.7–32.3)	< 0.001*
SBP (mmHg), mean ± SD	117.5 ± 16.6	110.9 ± 15.4	**0.012**
SBP percentile, median (25–75p)	97.5 (75.5–99)	93.5 (50.5–98.7)	0.042*
DBP (mmHg), mean ± SD	77.1 ± 16.9	71.1 ± 12.7	**0.020**
DBP percentile, median (25–75p)	96.5 (75–99)	91 (70.5–98.5)	0.065*
Heart rate (beats per minute, bpm), mean ± SD	99 ± 15	101 ± 16	0.215
IVC diameter max (cm), mean ± SD	0.84 ± 0.32	0.89 ± 0.32	0.266
IVC diameter min (cm), mean ± SD	0.62 ± 0.28	0.49 ± 0.21	**0.001**
IVC collapsibility index (%), mean ± SD	26.3 ± 10.01	44.4 ± 9.4	** < 0.001**
IJV diameter (cm), systole, mean ± SD	0.66 ± 0.26	0.70 ± 0.26	0.514
IJV diameter (cm), diastole, mean ± SD	0.51 ± 0.23	0.47 ± 0.18	0.363
Total B-line number, median (25–75p)	22 (15–27.7)	11.5 (10–16)	< 0.001*
Total body water, BIS, (L), median (25–75p)	14.8 (10.2–18.7)	14.9 (10.2–17.9)	0.712*
ECW (L), median (25–75p)	6.2 (4.4–8.93)	6.4 (4.3–8.8)	0.305*
ICW (L), mean ± SD	8.01 ± 2.66	8.0 ± 2.69	0.942
Fluid overload (L) BIS, mean ± SD	0.52 ± 0.64	0.43 ± 0.73	0.325
Fluid overload (%) BIS, mean ± SD	15.02 ± 11.42	7.48 ± 9.36	** < 0.001**
Fluid overload, by dry weight (L), median (25–75p)	0.8 (0.42–1.3)	0.4 (0–0.85)	< 0.001*
Fluid overload, by dry weight (%), median (25–75p)	3.15 (1.44–6.12)	1.25 (0–3.35)	< 0.001*

*DBP*, diastolic blood pressure; *ECW*, extracellular water; *ICW*, intracellular water; *IJV*, internal jugular vein; *IVC*, inferior vena cava; *SBP*, systolic blood pressure; *SD*, standard deviation. *Asterisked comparisons: Wilcoxon test; others: paired *t*-test

### Correlation analysis between ultrasound measurements and other parameters

**Fig. 1:**
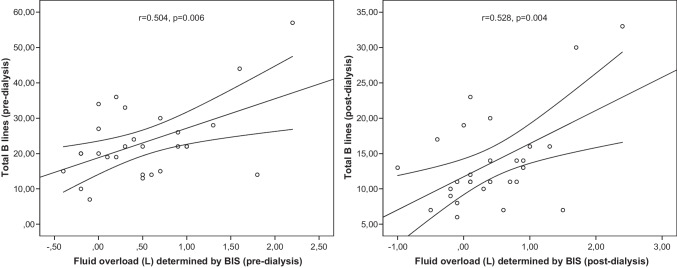
Correlation between pre- and post-dialysis total number of B-lines and fluid overload (L) determined using BIS

A moderate positive correlation was found between the total number of B-lines and fluid overload (L) measured by BIS both pre-dialysis (*r* = 0.504, *p* = 0.006) and post-dialysis (*r* = 0.528, *p* = 0.004) (Fig. 1). The pre-dialysis fluid overload (%) (*r* = 0.994, *p* < 0.001) and post-dialysis fluid overload (%) (*r* = 0.995, *p* < 0.001) measured using BIS showed a strong positive correlation with fluid overload (%) calculated based on dry weight. However, no significant correlation was observed between fluid overload measured using BIS and the IVC collapsibility index. The total number of B-lines showed a statistically significant moderate correlation (*r* = 0.380, *p* = 0.046) with the percentage of pre-dialysis fluid overload determined using BIS. In contrast, no significant correlation was found after dialysis (Table 2). The total number of B-lines was not correlated with systolic blood pressure percentile (*r* = − 0.065, *p* = 0.793), diastolic blood pressure percentile (*r* = − 0.087, *p* = 0.685) and IVC collapsibility index (*r* = − 0.326, *p* = 0.090).

**Table 2:** Correlation of fluid overload (%) determined using BIS with other parameters

	Pre-dialysis fluid overload (%) determined using BIS	Post-dialysis fluid overload (%) determined using BIS
Fluid overload by dry weight (%)	*r* = 0.994 *p* < 0.001	*r* = 0.995 *p* < 0.001
Total number of B-lines	*r* = 0.380 *p* = 0.046	*r* = 0.068 *p* = 0.729
IVC collapsibility index	*r* = −0.001 *p* = 0.994	*r* = 0.054 *p* = 0.784

*BIS*, bioimpedance spectroscopy; *IVC*, inferior vena cava

**Fig. 2:**
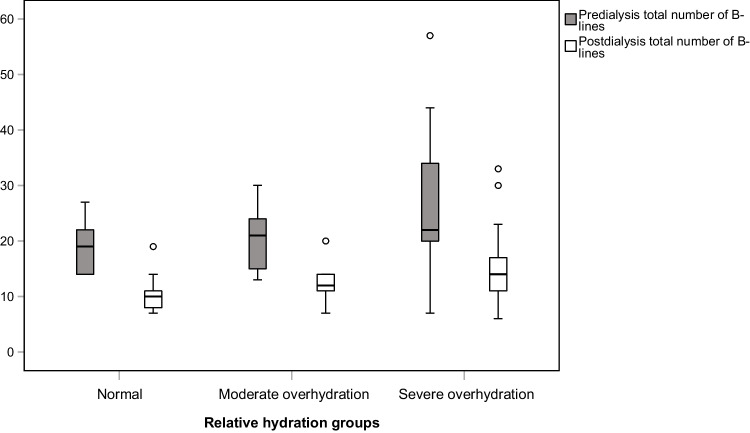
Boxplot showing the pre- and post-dialysis total number of B-lines among different hydration groups

No significant correlation was found between the total number of B-lines and the amount of ultrafiltrate (in mL and mL/kg) (*r* = 0.133, *p* = 0.499 and *r* = 0.117, *p* = 0.554, respectively). Similarly, no significant correlation was observed between the IVC collapsibility index, both pre-dialysis (*r* = − 0.139, *p* = 0.481 and *r* = − 0.151, *p* = 0.442) and post-dialysis (*r* = − 0.166, *p* = 0.400 and *r* = − 0.177, *p* = 0.368), and the amount of ultrafiltrate (in mL and mL/kg). A significant reduction in the post-dialysis total number of B-lines was observed across all hydration groups (*p* < 0.001). The most significant reduction in B-lines was observed in the severe overhydration group (Fig. 2). Supporting this, a moderate positive correlation was found between the difference in pre- and post-dialysis total number of B-lines and the relative overhydration percentage (*r* = 0.472, *p* = 0.011).

### Prediction of fluid overload using lung ultrasound B-lines

The AUC for the total number of B-lines in predicting severe overhydration assessed using BIS was 0.685 (95% confidence interval (CI): 0.472–0.897; *p* = 0.097). For predicting both moderate and severe overhydration using BIS, the AUC for the total number of B-lines was 0.673 (95% CI: 0.470–0.875; *p* = 0.147). Additionally, the AUC for the total number of B-lines in predicting overhydration assessed by weight was 0.740 (95% CI: 0.400–1.000; *p* = 0.181).

## Discussion

This study aimed to evaluate the fluid status in pediatric patients on peritoneal dialysis using novel methods such as lung ultrasound and BIS and to compare these with traditional clinical assessments. Our findings highlight the potential utility of these advanced techniques in identifying and monitoring fluid overload, particularly in pre- and post-dialysis states.

A study involving 13 dialysis patients found a significant linear correlation between fluid overload by weight and the total number of B-lines, while no significant correlation was observed with BIS, systolic blood pressure, or IVC collapsibility index [13]. In a subsequent study, the same author performed 142 lung ultrasound evaluations in 23 pediatric dialysis patients, showing a linear correlation between B-line score and fluid overload assessed by weight [14]. Similarly, our study showed a significant moderate correlation between the total number of B-lines and fluid overload assessed by weight and BIS. Additionally, the total number of B-lines was not correlated with systolic and diastolic blood pressure percentiles or IVC collapsibility index. A study performed in our center, which evaluated only pediatric hemodialysis patients, reported a strong correlation between fluid overload measured using BIS and the total number of B-lines [10]. Another study of 27 adult patients on peritoneal dialysis reported no significant correlation between the BIS values of fluid overload and the total number of B-lines, but N-terminal pro-brain natriuretic peptide (NT-proBNP) was correlated with both the total number of B-lines and BIS values of fluid overload [9]. In addition, Lučič Šrajer et al. reported a statistically significant correlation between the number of B-lines and NT-proBNP levels [15]. The studies including both hemodialysis and peritoneal dialysis patients demonstrated a significant correlation between the total number of B-lines and fluid overload determined using BIS. The results vary across studies probably due to various factors, including the number of patients on different types of dialysis, sample size, demographic and clinical characteristics of the study populations, and methodological variations such as differences in device sensitivity or protocols.

A pioneering study on fluid overload assessment in pediatric patients on hemodialysis showed an increase in IVC diameter and a decrease in collapsibility index due to fluid overload [16]. In the following years, additional studies were published evaluating the efficacy of IVC diameter and IVC collapsibility index in assessing fluid overload in pediatric dialysis patients [10, 13, 14, 17–19]. For instance, a study involving 16 patients on peritoneal dialysis and 9 on hemodialysis demonstrated a significant increase in the IVC collapsibility index values after hemodialysis, as well as after a 6-h dwell in the peritoneal dialysis group [18]. The data of the present study showed that the IVC collapsibility index significantly increased in patients after dialysis, even with a 2-h dwell time. However, no significant correlation was observed between the IVC collapsibility index and fluid overload assessed by both weight and BIS. Similarly, a previous study reported that IVC measurements were not reliable for assessing fluid overload in children undergoing hemodialysis because no correlation was found between IVC measurements and extracellular fluid volume assessed using BIS [19]. In a recent study involving 60 pediatric patients on hemodialysis, the IVC collapsibility index was evaluated to assess fluid overload, and it was found that the IVC diameter significantly decreased after dialysis, while the IVC collapsibility index significantly increased. The authors also concluded that lung B-lines outperformed IVC measurements and BIS in detecting subclinical volume overload in pediatric hemodialysis patients [20]. Several studies have demonstrated an increase in the IVC collapsibility index following ultrafiltration in dialysis. However, the reliability of the IVC collapsibility index in predicting fluid overload in pediatric patients on peritoneal dialysis has not been established. In our study, the IVC collapsibility index was not found to be a reliable method for assessing fluid overload in pediatric patients on peritoneal dialysis. One of the limitations of this technique, as previously reported, is that it is not suitable for infants and young children, as well as for those who are unable to cooperate with breath-holding during the procedure [3]. Moreover, obtaining adequate images is challenging, even for highly trained observers, making it less practical as a bedside tool. These factors likely contribute to the limited adoption of the IVC collapsibility index in clinical practice, particularly in pediatric populations.

The amount of ultrafiltrate did not correlate with the IVC collapsibility index, which is consistent with previous findings in pediatric patients [18]. Similarly, no correlation was found between ultrafiltrate volume and total number of B-lines. Nevertheless, the greatest reduction in the total number of B-lines after dialysis occurred in patients with severe overhydration, indicating the most significant improvement in pulmonary congestion in this group.

In the study by Tan et al., the AUC values for B-lines, overhydration, and relative overhydration (%) determined using lung ultrasound were 0.697 (95% CI 0.586–0.808) and 0.713 (95% CI 0.603–0.822), respectively. They concluded that detecting B-lines might provide clinically useful information in assessing hydration status and diagnosing fluid overload in patients on dialysis [21]. In contrast, our ROC analysis did not provide statistically significant AUC values for the number of B-lines in predicting fluid overload as defined by either BIS or weight-based criteria. While a positive correlation between B-lines and fluid overload was observed, their discriminative performance in our cohort was limited. This inconsistency may be attributed to differences in dialysis modality (peritoneal vs. hemodialysis), age group, or sample size, among other potential contributing factors.

Our study had some limitations. Firstly, the limited number of pediatric patients on peritoneal dialysis included in our study restricted the generalizability of our findings. Furthermore, our results could not be compared with previous findings due to the limited number of similar studies including only pediatric patients on chronic peritoneal dialysis. Despite these limitations, we believe our study contributes to the existing data and highlights the potential role of lung ultrasound and BIS in evaluating fluid status and related complications in pediatric patients undergoing peritoneal dialysis.

## Conclusions

This study was unique in evaluating multiple novel methods to assess both pre-dialysis fluid overload and volume changes following ultrafiltration post-dialysis in pediatric patients on peritoneal dialysis. We used a combination of lung ultrasound and the IVC collapsibility index methods to predict fluid overload. Our findings may serve as a foundation for future studies aimed at identifying fluid overload in pediatric patients on peritoneal dialysis. Further randomized controlled trials involving a larger number of pediatric patients on peritoneal dialysis are needed to validate the reliability of using lung ultrasound and the IVC collapsibility index together for assessing pre-dialysis fluid overload and determining dry weight.

## Supplementary Information

Below is the link to the electronic supplementary material.

Graphical abstract (PDF 100 KB)

## Data Availability

The data that supports the findings of this study are available from the corresponding author on reasonable request.
