# Supplementary material for: Unmasking fluid overload in children on peritoneal dialysis: a multimodal diagnostic approach
Source: Pediatr Nephrol. 2025 Jul 3;40(11):3505–11. doi: 10.1007/s00467-025-06825-y (PMC12484372; doi:10.1007/s00467-025-06825-y)
Supplement: Supplementary file 1 — Graphical abstract (PDF 100 KB) [file 467_2025_6825_MOESM1_ESM.pptx]

## Slide 1
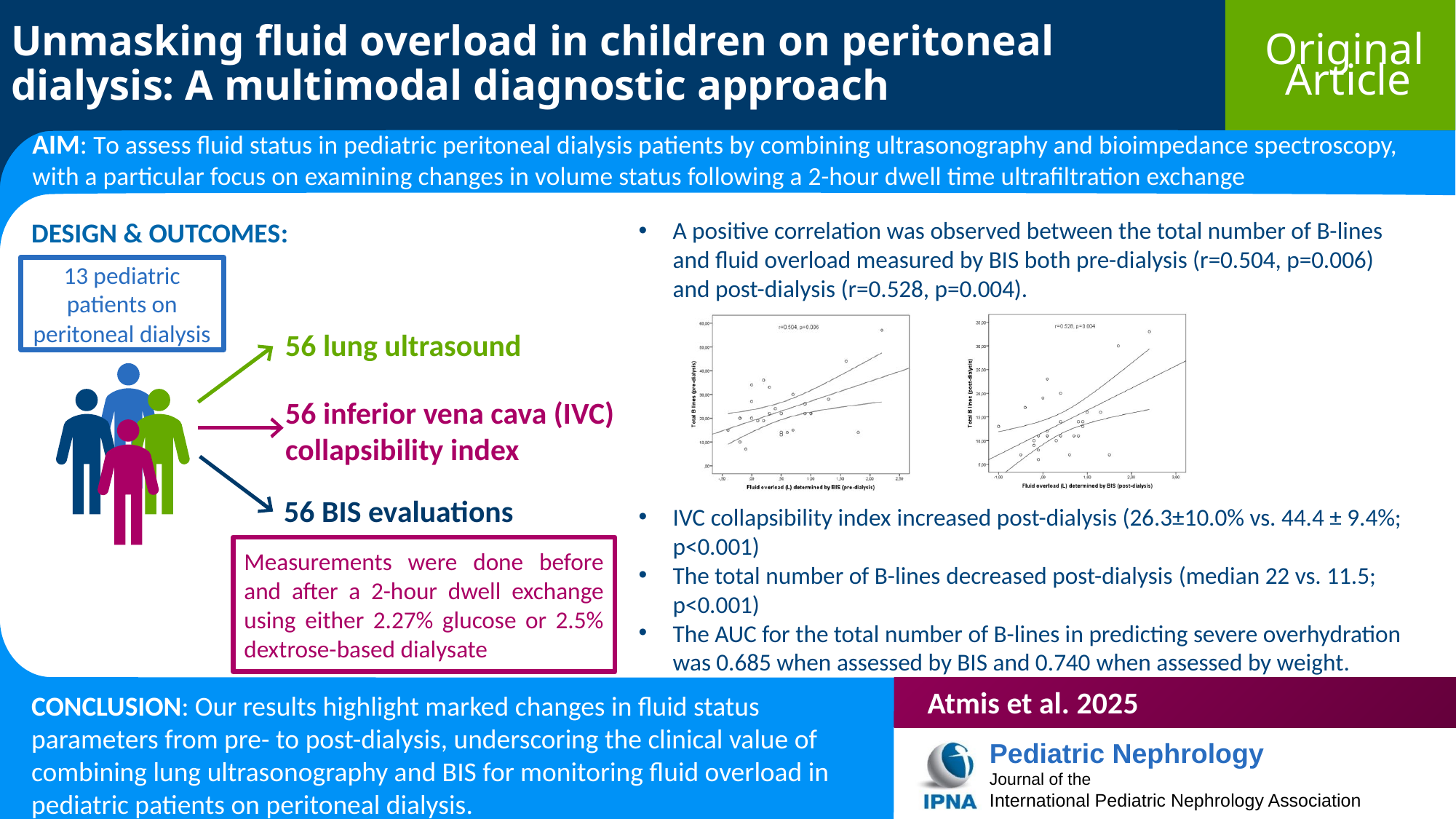

Unmasking fluid overload in children on peritoneal dialysis: A multimodal diagnostic approach
AIM: To assess fluid status in pediatric peritoneal dialysis patients by combining ultrasonography and bioimpedance spectroscopy, with a particular focus on examining changes in volume status following a 2-hour dwell time ultrafiltration exchange
A positive correlation was observed between the total number of B-lines and fluid overload measured by BIS both pre-dialysis (r=0.504, p=0.006) and post-dialysis (r=0.528, p=0.004).
DESIGN & OUTCOMES:
13 pediatric patients on peritoneal dialysis
56 lung ultrasound
56 inferior vena cava (IVC) collapsibility index
56 BIS evaluations
IVC collapsibility index increased post-dialysis (26.3±10.0% vs. 44.4 ± 9.4%; p<0.001)
The total number of B-lines decreased post-dialysis (median 22 vs. 11.5; p<0.001)
The AUC for the total number of B-lines in predicting severe overhydration was 0.685 when assessed by BIS and 0.740 when assessed by weight.
Measurements were done before and after a 2-hour dwell exchange using either 2.27% glucose or 2.5% dextrose-based dialysate
Atmis et al. 2025
CONCLUSION: Our results highlight marked changes in fluid status parameters from pre- to post-dialysis, underscoring the clinical value of combining lung ultrasonography and BIS for monitoring fluid overload in pediatric patients on peritoneal dialysis.
